# New Urinary Tests

**Published:** 1915-03

**Authors:** 


**New Urinary Tests**, Klein and Walker, Post Graduate, September, 1914.

Skatol Test. About 4 or 5 c.c. of mixed Fehling's solution is heated. To this 2 c.c. or 3 c.c. of urine is added and the mixture heated. Now add to this mixture 2 to 5 c.c. of a one tenth aqueous solution of iodine. If acetone is present, especially in large quantities, the odor of iodoform first predominates and after a few minutes the smell of skatol is distinctly identified. This odor is more or less permanent. If diabetic urine is treated for skatol, the heat should be diminished or a modified Fehling solution used.

Oxidase or Kidney Test. Add 1 c.c. to 3 c.c. of ammonia molybdate to 3 c.c. or more of undiluted urine. A precipitate forms. Add gradually to this a 25% solution of ammonia sufficient to dissolve the precipitate and to obtain a blue-colored solution of reduced molybdic acid. If a kidney lesion as Bright's is present the solution loses its blue color to a greater or less degree. The degree and rapidity of the change denotes the extent of kidney involvement or the severity of the lesion.

Pancreas Test. This is similar to the kidney test, using, however, only ammonium molybdate. After the precipitate has settled, a bluish green color of the supernatant solution, gradually fading, shows that the pancreas is in some degree affected.

Selenium Test. This test may be applied to the patients using selenium for malignancy and will indicate a point of saturation. To 10 c.c. or 15 c.c. of urine 5 c.c. of freshly prepared stannous chloride is added and an equal volume of ether or benzol. Shake the mixture well. The ether or benzol being much lighter than the urine will rise to the top. The selenium if present will be seen at a distinct red zone at the junction of the two liquids.

